# The impact of artificial intelligence on learner–instructor interaction in online learning

**DOI:** 10.1186/s41239-021-00292-9

**Published:** 2021-10-26

**Authors:** Kyoungwon Seo, Joice Tang, Ido Roll, Sidney Fels, Dongwook Yoon

**Affiliations:** 1 Department of Applied Artificial Intelligence, Seoul National University of Science and Technology, 232 Gongneung-ro, Gongneung-dong, Nowon-gu, Seoul, 01811 Korea; 2 Department of Computer Science, The University of British Columbia, Vancouver, Canada; 3 Faculty of Education in Science and Technology, Technion-Israel Institute of Technology, Haifa, Israel; 4 Department of Electrical and Computer Engineering, The University of British Columbia, Vancouver, Canada

**Keywords:** Artificial intelligence, Boundary, Learner–instructor interaction, Online learning, Speed dating

## Abstract

Artificial intelligence (AI) systems offer effective support for online learning and teaching, including personalizing learning for students, automating instructors’ routine tasks, and powering adaptive assessments. However, while the opportunities for AI are promising, the impact of AI systems on the culture of, norms in, and expectations about interactions between students and instructors are still elusive. In online learning, learner–instructor interaction (inter alia, communication, support, and presence) has a profound impact on students’ satisfaction and learning outcomes. Thus, identifying how students and instructors perceive the impact of AI systems on their interaction is important to identify any gaps, challenges, or barriers preventing AI systems from achieving their intended potential and risking the safety of these interactions. To address this need for forward-looking decisions, we used Speed Dating with storyboards to analyze the authentic voices of 12 students and 11 instructors on diverse use cases of possible AI systems in online learning. Findings show that participants envision adopting AI systems in online learning can enable personalized learner–instructor interaction at scale but at the risk of violating social boundaries. Although AI systems have been positively recognized for improving the quantity and quality of communication, for providing just-in-time, personalized support for large-scale settings, and for improving the feeling of connection, there were concerns about responsibility, agency, and surveillance issues. These findings have implications for the design of AI systems to ensure explainability, human-in-the-loop, and careful data collection and presentation. Overall, contributions of this study include the design of AI system storyboards which are technically feasible and positively support learner–instructor interaction, capturing students’ and instructors’ concerns of AI systems through Speed Dating, and suggesting practical implications for maximizing the positive impact of AI systems while minimizing the negative ones.

## Introduction

The opportunities for artificial intelligence (AI) in online learning and teaching are broad (Anderson et al., 1985; Baker, 2016; Roll et al., 2018; Seo et al., 2020b; VanLehn, 2011), ranging from personalized learning for students and automation of instructors’ routine tasks to AI-powered assessments (Popenici & Kerr, 2017). For example, AI tutoring systems can provide personalized guidance, support, or feedback by tailoring learning content based on student-specific learning patterns or knowledge levels (Hwang et al., 2020). AI teaching assistants help instructors save time answering students’ simple, repetitive questions in online discussion forums, and instead instructors can dedicate their saved time to higher-value work (Goel & Polepeddi, 2016). AI analytics allows instructors to understand students’ performance, progress, and potential by decrypting their clickstream data (Roll & Winne, 2015; Fong et al., 2019; Seo et al., 2021; Holstein et al., 2018).

While the opportunities for AI are promising, students and instructors may perceive the impact of AI systems negatively. For instance, students may perceive indiscriminate collection and analysis of their data through AI systems as a privacy breach, as illustrated by the Facebook–Cambridge Analytica data scandal (Chan, 2019; Luckin, 2017). The behavior of AI agents that do not take into account the risk of data bias or algorithmic bias can be perceived by students as discriminatory (Crawford & Calo, 2016; Murphy, 2019). Instructors worry that relying too much on AI systems might compromise the student’s ability to learn independently, solve problems creatively, and think critically (Wogu et al., 2018). It is important to examine how students and instructors perceive the impact of AI systems in online learning environments (Cruz-Benito et al., 2019).

The AI in Education (AIEd) community is increasingly exploring the impact of AI systems in online education. For example, Roll and Wylie (2016) call for more involvement of AI systems in the communication between students and instructors, and in education applications outside school context. At the same time, Zawacki-Richter and his colleagues (2019) conducted a systematic review of AIEd publications from 2007 to 2018 and as a result found a lack of critical reflection of the ethical impact and risks of AI systems on learner–instructor interaction. Popenici and Kerr (2017) investigated the impact of AI systems on learning and teaching, and uncovered potential conflicts between students and instructors, such as privacy concerns, changes in power structures, and excessive control. All of these studies called for more research into the impact of AI systems on learner–instructor interaction, which will help us identify any gaps, issues, or barriers preventing AI systems from achieving their intended potential.

Indeed, learner–instructor interaction plays a crucial role in online learning. Kang and Im (2013) demonstrated that factors of learner–instructor interaction, such as communication, support, and presence, improve students’ satisfaction and learning outcomes. The learner–instructor interaction further affects students’ self-esteem, motivation to learn, and confidence in facing new challenges (Laura & Chapman, 2009). Less is known, however, about how introducing AI systems in online learning will affect learner–instructor interaction. Guilherme (2019, p. 7) predicted that AI systems would have “a deep impact in the classroom, changing the relationship between teacher and student.” More work is needed to understand how and why various forms of AI systems affect learner–instructor interaction in online learning (Felix, 2020).

Considering the findings in the literature and the areas for further research, the present study aimed to identify how students and instructors perceive the impact of AI systems on learner–instructor interaction in online learning. To this end, we used Speed Dating, a design method that allows participants to quickly interact with and experience the concepts and contextual dimensions of multiple AI systems without any technical implementation (Davidoff et al., 2007). In Speed Dating, participants are presented with various hypothetical scenarios via storyboards while researchers conduct interviews to understand the participants’ immediate reactions (Zimmerman & Forlizzi, 2017). These interviews provided rich opportunities to understand the way students and instructors perceive the impact of AI systems on learner–instructor interaction and the boundaries beyond which AI systems are perceived as “invasive.”

The study offers several unique contributions. First, as part of the method, we designed storyboards that can be used to facilitate further research on AI implications for online learning. Second, the study documents the main promises and concerns of AI in online learning, as perceived by both students and instructors in higher education. Last, we identify practical implications for the design of AI-based systems in online learning. These include empahses on explainability, human-in-the-loop, and careful data collection and presentation.

This paper is organized as follows. The next section provides the theoretical framework and background behind this research paper by describing the main aspects of the learner–instructor interaction and AI systems in education. “Materials and methods” section is related to the methodological approach followed in this research and describes the storyboards used to collect data, the participants, the study procedure, and the performed qualitative analysis. “Findings” section shows the results obtained and the main findings related to the research question. Finally, “Discussion and conclusion” section provides an overview of the study’s conclusions, limitations, and future research.

## Background

This paper explores the impact of AI systems on learner–instructor interaction in online learning. We first proposed a theoretical framework based on studies on learner–instructor interaction in online learning. We then reviewed the AI systems currently in use in online learning environments.

### Theoretical framework

Interaction is paramount for successful online learning (Banna et al., 2015; Nguyen et al., 2018). Students exchange information and knowledge through interaction and construct new knowledge from this process (Jou et al., 2016). Moore (1989) classified these interactions in online learning into three types: learner–content, learner–learner, and learner–instructor. These interactions help students become active and more engaged in their online courses (Seo et al., 2021; Martin et al., 2018), and by doing so strengthen their sense of community which is essential for the continuous usage of online learning platforms (Luo et al., 2017).

Martin and Bolliger (2018) found that the learner–instructor interaction is the most important among Moore’s three types of interactions. Instructors can improve student engagement and learning by providing a variety of communication channels, support, encouragement, and timely feedback (Martin et al., 2018). Instructors can also enhance students’ sense of community by engaging and guiding online discussions (Shackelford & Maxwell, 2012; Zhang et al., 2018). Collectively, learner–instructor interaction has a significant impact on students’ satisfaction and achievement in online learning (Andersen, 2013; Kang & Im, 2013; Walker, 2016).

The five-factor model of learner–instructor interaction offers a useful lens for interpreting interactions between students and the instructor in online learning (see Table 1; Kang, 2010). Robinson et al. (2017) found that communication and support are key factors of the learner–instructor interaction for designing meaningful online collaborative learning. Richardson et al. (2017) added that the perceived presence during learner–instructor interaction positively influences student motivation, satisfaction, learning, and retention in online courses. Kang and Im (2013) synthesized these findings by showing that communication, support, and presence are the three most important factors in improving students’ achievement and satisfaction over other factors. Thus, in this study, we focused on communication, support, and presence between students and instructors.

**Table 1:** The five-factor model of learner–instructor interaction in online learning environments, adapted from Kang and Im (2013)

Factor of learner–instructor interaction	Definition
Communication	Instructional communication (Q & A) between learners and the instructor about topics directly related to learning contents
Support	Instructional management by the instructor, including supporting learning materials and providing feedbacks directly related to learning contents
Presence	Perceived connectivity between students and instructors during the online learning process
Guidance	Guidance by the instructor through providing encouragement and positive reactions that are not directly related to learning contents
Social intimacy	Social interaction by the instructor, such as introduction, greetings, and exchange of personal information that are not directly related to learning contents

AI systems are likely to affect the way learner–instructor interaction occurs in online learning environments (Guilherme, 2019). If students and instructors have strong concerns about the impact of AI systems on their interactions, then they would not use such systems, in spite of perceived benefits (Felix, 2020). To the best of our knowledge, the impact of AI systems on learner–instructor interaction has limited empirical studies, and Misiejuk and Wasson (2017) have called for more work on this.

### Artificial intelligence in online learning

There are a variety of AI systems that are expected to affect learner–instructor interaction in online learning. For example, Goel and Polepeddi (2016) developed an AI teaching assistant named Jill Watson to augment the instructor’s communication with students by autonomously responding to student introductions, posting weekly announcements, and answering routine, frequently asked questions. Perin and Lauterbach (2018) developed an AI scoring system that allows faster communication of grades between students and the instructor. Luckin (2017) showed AI systems that support both students and instructors by providing constant feedback on how students learn and the progress they are making towards their learning goals. Ross et al. (2018) developed online adaptive quizzes to support students by providing learning contents tailored to each student’s individual needs, which improved student motivation and engagement. Heidicker et al. (2017) showed that virtual avatars allow several physically separated users to collaborate in an immersive virtual environment by increasing sense of presence. Aslan and her colleagues (2019) developed AI facial analytics to improve instructors’ presence as a coach in technology-mediated learning environments. When looking at these AI systems, in-depth insight into how students and instructors perceive the AI’s impact is important (Zawacki-Richter et al., 2019).

The recent introduction of commercial AI systems for online learning has demonstrated the complex impact of AI on learner–instructor interaction. For instance, Proctorio (Proctorio Inc., USA), a system that aims to prevent cheating by monitoring students and their computer screens during an exam, seems like a fool-proof plan to monitor students in online learning, but students complain that it increases their test-taking anxiety (McArthur, 2020). The idea of being recorded by Proctorio distracts students and creates an uncomfortable test-taking atmosphere. In a similar vein, although Squirrel AI (Squirrel AI Learning Inc., China) aims to provide adaptive learning by adjusting itself automatically to the best method for an individual student, there is a risk that this might restrict students’ creative learning (Beard, 2020). These environments have one thing in common: Unlike educational technologies that merely mediate interactions between instructors and students, AI systems have more autonomy in the way in which it interprets data, infers learning, and at times, takes instructional decisions.

In what follows, we describe Speed Dating with storyboards, an exploratory research method that allows participants to quickly experience different forms of AI systems possible in the near future, to examine the impact of those systems on learner–instructor interaction (“Materials and methods”). Findings offer new insights on students’ and instructors’ boundaries, such as when AI systems are perceived as “invasive” (“Findings”). Lastly, we discuss how our findings provide implications for future AI systems in online learning (Discussion and conclusion).

## Materials and methods

The goal of this study is to gain insight on students’ and instructors’ perception of the impact of AI systems on learner–instructor interaction (inter alia, communication, support, and presence; Kang & Im, 2013) in online learning. The study was conducted amid the COVID-19 pandemic, thus students and instructors have heightened awareness about the importance of online learning and fresh experiences from the recent online courses. Our aim was not to evaluate specific AI technologies, but instead, to explore areas where AI systems positively contribute to learner–instructor interaction and where more attention is required.

We used Speed Dating with storyboards, an exploratory research method that allows participants to experience a number of possible AI systems in the form of storyboards, to prompt participants to critically reflect on the implications of each AI area (Zimmerman & Forlizzi, 2017). Exposure to multiple potential AI areas that are likely to be available in the future helps participants to shape their own perspectives and to evaluate the AI systems in a more nuanced way (Luria et al., 2020). We first created a set of eleven four-cut storyboards for the comprehensive and diverse use cases of possible AI systems in online learning (see “Creating storyboards” section), and then used these storyboards to conduct Speed Dating with student and instructor participants (see “Speed dating” section). Overall, we address the following research question:

- How do students and instructors perceive the impact of AI systems on learner–instructor interaction (inter alia, communication, support, and presence) in online learning?

### Creating storyboards

To create AI system storyboards which are technically feasible and positively support learner–instructor interaction, we ran an online brainwriting activity (Linsey & Becker, 2011) in which we asked a team of designers to come up with scenarios about possible AI systems in online learning. We recruited six designers from our lab (four faculty members and two PhD candidates) with an average of 15.4 years (SD = 4.7 years) of design experience in human–computer interaction (HCI). Each team member wrote down scenarios using a Google Slides file and then passed it on to another team member. This process was repeated four times until all designers agreed that the scenarios of AI systems were technically feasible and supported learner–instructor interaction in online learning.

As initial scenarios were made by HCI designers, in order to validate their technical feasibility and positive impact on learner–instructor interaction, we enacted additional interviews with six AI experts with an average of 10.8 years (SD = 7.8 years) of research experience and 8 years (SD = 6.2 years) of teaching experience (see Appendix A , Table 7, for details). The first two authors conducted semi-structured interviews with AI experts using a video conferencing platform (i.e., Zoom). We showed each scenario to AI experts and asked the following questions: “Can you improve this scenario to make it technically feasible?” and “Can you improve this scenario to have a positive impact on learner–instructor interaction based on your own online teaching experience?” After showing all the scenarios, the following question was asked: “Do you have any research ideas that can be used as a new scenario?” The scenario was modified to reflect the opinions of AI experts and AIEd literature. The interviews lasted around 41 min on average (SD = 7.3 min). Each AI expert was compensated 100 Canadian dollars for their time. The process was cleared by the Research Ethics Board.

As shown in Table 2, we ended up with 11 scenarios which support learner–instructor interaction (i.e., communication, support, and presence) in online learning. Scenarios were categorized by the first two authors with reference to the learner–instructor interaction factors as defined in Table 1 (see “Theoretical framework” section). For example, although the AI Teaching or Grading Assistant scenarios could be considered systems of support for the instructor, “support” within the learner–instructor interaction framework refers to support for the student. Therefore, since the scenarios illustrate increased or expedited communication between students and instructors rather than direct support for students, AI Teaching and Grading Assistant scenarios are categorized as systems for communication. We note that these categories are not definitive, and scenarios may have interleaving aspects of several learner–instructor interaction factors. However, the final categories in Table 2 refer to the factors that best define the respective scenarios.

**Table 2:** Factors of learner–instructor interaction, scenario titles, and scenario summaries

ID	Factor of learner–instructor interaction	Scenario title	Scenario summary
1	Communication	AI Teaching Assistant (Goel & Polepeddi, 2016)	AI answers student questions before, during, or after online courses based on answers to questions gathered in previous courses
2		AI Companion (Woolf et al., 2010)	AI emotionally supports students who are concerned about their grades and workload, and provides assistance when students use language related to self-destructive behavior
3		AI Grading Assistance (Perin & Lauterbach, 2018)	AI helps TAs quickly grade assignments by offering suggestions that they should choose to accept or change for each question
4		AI Peer Review	AI normalizes peer review grades by keeping students’ holistic profiles in mind, and by comparing each students’ history of peer reviews with others as well as with peer reviews from previous iterations of the course
5	Support	AI Analytics (Luckin, 2017)	AI provides an analysis of students’ clickstream, quiz, login/logout, and eye-tracking data to instructors
6		Intelligent Suggestions (Luckin, 2017)	AI suggests study materials and strategies to students based on an analysis of students' clickstream and quiz performance data
7		AI Group Project Organizer	AI helps write meeting minutes using speech recognition, suggests action plans from group discussions through text summarization, and gives editing tips based on assignment data from previous iterations of the course
8		Adaptive Quiz (Ross et al., 2018)	AI provides students with a personalized set of exercise problems that suits their level of knowledge
9	Presence	Virtual Avatar (Heidicker et al., 2017)	AI communicates facial expressions and body language without explicitly using a student’s camera feed through a virtual avatar
10		AI Breakout Room Matching	AI matches students in breakout rooms in a way that optimizes discussion by analyzing microphone data (e.g., frequency, length and tone)
11		AI Facial Analytics (Aslan et al., 2019)	AI gauges students’ emotions without sharing videos, and notifies the instructor in real-time when a specific student seems especially distressed or unengaged

Seven scenarios (Scenarios 1, 3, 5, 6, 8, 9, and 11) have well reflected the state-of-the-art AI systems that were identified in “Artificial intelligence in online learning” section. The following four scenarios were created based on research ideas from AI experts: AI Companion (Scenario 2), AI Peer Review (Scenario 4), AI Group Project Organizer (Scenario 7), and AI Breakout Room Matching (Scenario 10). These 11 final scenarios were not to exhaust all AI systems in online learning or to systematically address all topics, but rather to probe a range of situations that shed light on the realities that present themselves with the utilization of AI systems in online learning.

**Fig. 1:**
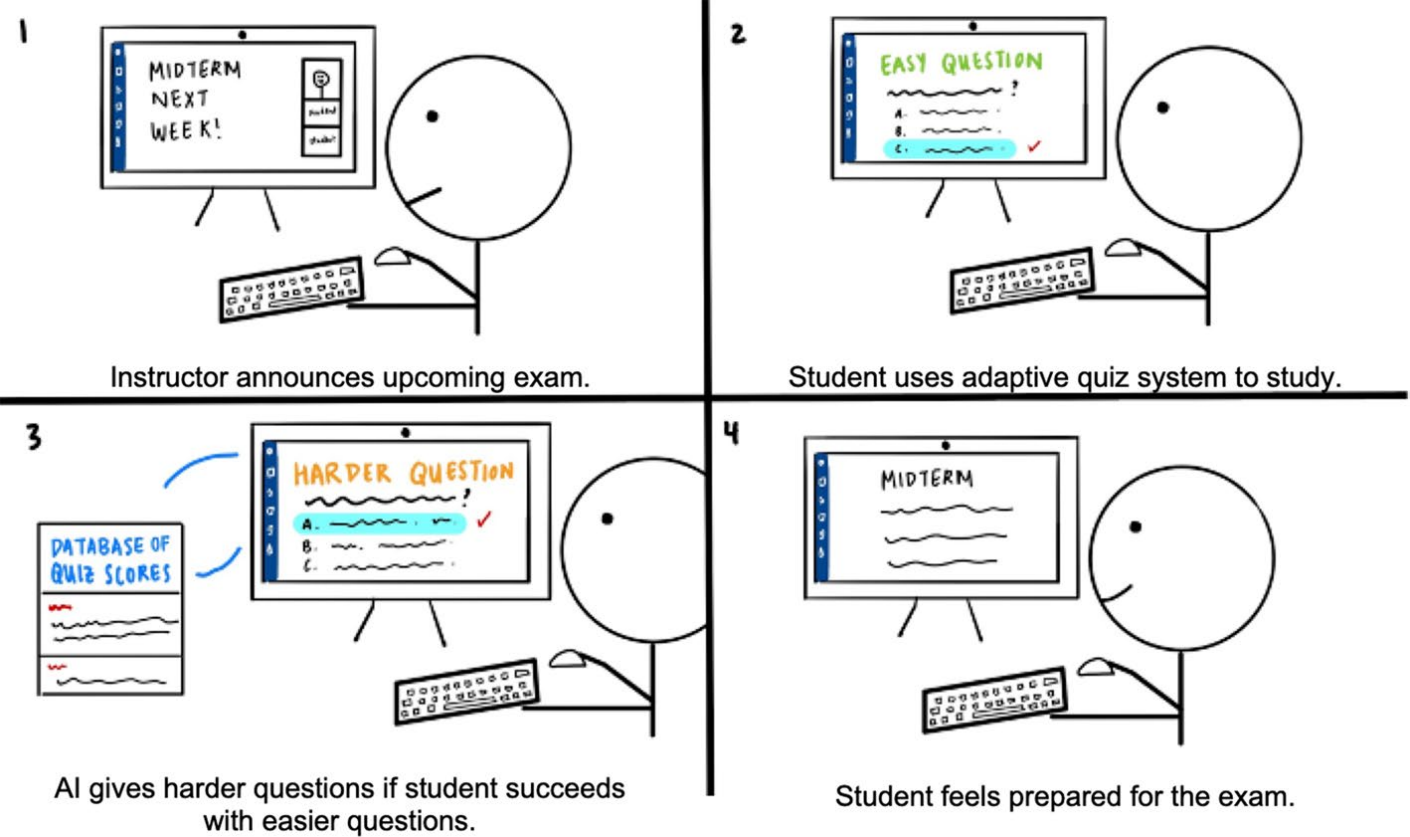
A storyboard example of scenario 8, Adaptive Quiz in Table 2

We generated four-cut storyboards based on the scenarios in Table 2. Figure 1 shows an illustrated example of a storyboard detailing the scenario through captions. We stylized the characters in a single visual style and as flat cartoon shades in order to reduce gender and ethnic clues and enable participants to put themselves in the shoes of the characters in each storyboard (Truong et al., 2006; Zimmerman & Forlizzi, 2017). The full set of storyboards can be viewed at https://osf.io/3aj5v/?view_only=bc5fa97e6f7d46fdb66872588ff1e22e.

### Speed dating

#### Participants

Next, we conducted a Speed Dating activity with storyboards. We recruited 12 students (see Table 3) and 11 instructors (see Table 4) for a Speed Dating activity. For diversity, we recruited students from 11 different majors and recruited instructors from nine different subjects. Students and instructors had a minimum of three months of online learning or teaching experience due to the COVID-19 pandemic. Overall, students had at least one year of university experience and instructors had at least three years of teaching experience. We required students and instructors to have online learning and teaching experience respectively so as to control the expected and experienced norms of student-instructor interaction within online university classes. Conversely, we did not require participants to have knowledge of AI systems as we wanted their perspective on the intended human–AI interactions and their potential effects as illustrated. Previous studies showed that Speed Dating works well without any prior knowledge or experience with AI systems, so no special knowledge or experience was required to participate in this study (Luria et al., 2020; Zimmerman & Forlizzi, 2017). Each participant was compensated with 25 Canadian dollars for their time.

**Table 3:** Summary of the students’ information

ID	Major	Year level	Age	Gender
S1	Economics	4	21	M
S2	Biology	4	21	W
S3	Sociology	3	20	W
S4	Behavioural Neuroscience	4	20	W
S5	Psychology	4	21	W
S6	Computer Science	2	19	M
S7	Nursing	2	20	W
S8	Computer Engineering	3	21	M
S9	Business and Computer Science	4	21	W
S10	Computer Science	5	22	M
S11	Civil Engineering	4	20	M
S12	Philosophy	2	18	W

**Table 4:** Summary of the instructors’ information

ID	Teaching subject	Teaching experience	Average class size	Age	Gender
I1	Political Science	20 years	150	56	M
I2	Chinese Language and Culture	6 years	30	34	W
I3	Chinese Language and Culture	5 years	30	31	W
I4	Business Analytics	5 years	60	37	M
I5	Korean Language	17 years	30	42	W
I6	Chinese Language	12 years	25	45	W
I7	Occupational Therapy	45 years	50	51	W
I8	Physics	22 years	180	56	M
I9	Computer Science	27 years	170	52	M
I10	Computer Science	3 years	130	28	W
I11	Chemistry	14 years	430	37	M

#### Procedure

We conducted semi-structured interviews with participants using a video conferencing platform (i.e., Zoom). We designed the interview questions to capture how the participants perceive the AI systems illustrated in the storyboards (see Appendix B). Participants read each of the storyboards aloud and then expressed their perceptions of AI in online learning. Specifically, we asked participants to critically reflect on how incorporating the AI system into an online course would affect learner–instructor interaction and whether they would like to experience its effect. We also asked them to choose AI systems that would work well and which would not work well, to capture their holistic point of view regarding perceived affordances and drawbacks. The entire interview lasted around 50.9 min (SD = 10.7 min), with 3–5 min spent to share each storyboard and probe participants on its specific implications.

#### Data analysis

Each interview was audio recorded and transcribed for analysis. We used a Reflexive Thematic Analysis approach (Braun & Clarke, 2006; Nowell et al., 2017). After a period of familiarization with the interview data, the first two authors began by generating inductive codes with an initial round of semantic codes related to intriguing statements or phrases in the data. The two authors then coded each transcript by highlighting and commenting on data items through Google Docs, independently identifying patterns that arose through extended examination of the dataset. Any conflicts regarding such themes were resolved through discussion between the two authors. Later, through a deductive approach guided by the learner–instructor interaction factors adapted from Kang and Im (2013), data were coded and collated into themes in a separate word document. An example of our codes can be viewed at https://osf.io/3aj5v/?view_only=bc5fa97e6f7d46fdb66872588ff1e22e. We then utilized three iterative discussions with all five authors present that yielded recurrent topics and themes by organizing the data around significant thematic units; the final six major themes were derived from twelve codes. The themes, which describe the impact of AI systems, were as follows: (1) Quantity and Quality, (2) Responsibility, (3) Just-in-time Support, (4) Agency, (5) Connection, and (6) Surveillance. The findings below are presented according to these themes.

## Findings

The central theme of participants’ responses, which stood out repeatedly in our study, was that adopting AI systems in online learning can enable personalized learner–instructor interaction at scale but at the risk violating social boundaries. Participants were concerned that AI systems could create responsibility, agency, and surveillance issues in online learning if they violated social boundaries in each factor of learner–instructor interaction (i.e., communication, support, and presence). Table 5 summarizes the perceived benefits and concerns of students and instructors about the impact of AI systems on learner–instructor interaction, as noted with ( +) and ( −) respectively. Each quote outlines whether the response came from a student (“S”) or an instructor (“I”).

**Table 5:** Summary of the students’ and instructors’ perceptions of AI systems in online learning

Factor of learner–instructor interaction	The impact of AI systems	Students’ perceptions	Instructors’ perceptions
Communication	Quantity & Quality	( +) Students believe that the anonymity afforded by AI would make them less self-conscious and, as a result, allow them to ask more questions	( +) Instructors believe that AI could help answer simple, repetitive questions, which would allow them to focus on more meaningful communication with students
	Responsibility	( −) Students worry that AI could give unreliable answers and negatively impact their grades	( −) Instructors predicted conflicts between students and the instructor due to AI-based misunderstandings or misleadingness
Support	Just-in-time support	( +) Students believe that AI would support personalized learning experiences, particularly with studying and group projects	( +) Instructors believe AI could be effectively leveraged to help students receive just-in-time personalized support
	Agency	( −) Students perceived that canned and standardized support from AI might have a negative influence on their ability to learn effectively	( −) Instructors are wary of the fact that too much support from AI could take away students' opportunities for exploration and discovery
Presence	Connection	( +) Students believe that AI can address privacy concerns and support learner–instructor connections by providing social interaction cues without personal camera information	( +) Instructors believe that the addition of AI would help them become more aware of students’ needs
	Surveillance	( −) Students are uncomfortable with the measurement of their unconscious behavior, such as eye tracking or facial expression analysis, because it feels like surveillance	( −) Instructors were negative about relying on AI interpretation to understand students’ social interaction cues

( +) indicates perceived benefit and ( −) indicates perceived concern

### Communication

In online learning environments, communication refers to questions and answers between students and the instructor about topics directly related to learning contents, such as instructional materials, assignments, discussions, and exams (Kang & Im, 2013). Students and instructors expect AI systems will positively impact the quantity and quality of communication between them but bears the risk causing miscommunication and responsibility issues, as described below.

#### Quantity and quality

*Students believe that the anonymity afforded by AI would make them less self-conscious and, as a result, allow them to ask more questions*. In online learning environments, students are generally afraid to ask questions to their instructors during class, primarily because they “worry that someone already asked it” (S4) or “don't want to seem dumb by instructors or peers” (S10). Students perceive that the anonymity from both an AI Teaching Assistant (Scenario 1) and an AI Companion (Scenario 2) would make them “less afraid to ask questions” (S10), “wouldn't feel bad about wasting the professor's time” (S11), and would be “less distracting to class” (S12). Bluntly put, participant S11 stated: “If it’s a dumb question, I’ve got an AI to handle it for me. The AI won't judge me. The AI is not thinking like, wow, what an idiot.” S5 expanded on this idea, mentioning that asking questions to an AI removes self-consciousness that typically exists in instructional communications: “… you don’t feel like you’re bothering a person by asking the questions. You can’t really irritate an AI, so you can ask as many as you need to.” As a result, all 12 students answered that AI systems would nudge them to ask more questions in online learning.

*Instructors believe that AI could help answer simple, repetitive questions, which would allow them to focus on more meaningful communication with students*. Answering repetitive questions from students takes a huge amount of time (I11). Instructors reflected that the time saved from tedious tasks, such as answering administrative questions, could allow course teams to focus on more content-based questions (I10). Because an AI Teaching Assistant (Scenario 1) answers students’ repetitive questions and AI Grading Assistance (Scenario 3) and AI Peer Review (Scenario 4) enable fast feedback loops, instructors can communicate more meaningfully with students by helping to “focus more on new questions” (I6) or “use their time for more comprehensive or more irregular questions” (I4). As well-stated by I10: “I think it allows us time to have conversations that are more meaningful… in some ways you're choosing quality over quantity. The more time I have, the more time, I can do things like answer emails or answer things on Piazza, things that actually will communicate with the student.”

#### Responsibility

*Although students believe AI systems would improve the quantity and quality of instructional communication, they worry that AI could give unreliable answers and negatively impact their grades*. For example, S4 worried that “I just want to make sure it’s a really reliable source, because if the AI is answering questions from students, and then they’re going to apply that answer to the way they do their work in the future, and it might be marked wrong. Then it's hard to go to the instructor and say, oh, this answer was what was given to me, but you said it was wrong.” Most students (10 out of 12) feel like the lack of explainability of AI would make it hard to blame despite the fact that it may hold a position of responsibility in some situations, such as answering questions where its answers should be considered as truth. S9 said that “Whereas with AI and just intelligent systems that you don't fully understand the back end to in a sense, it’s harder to decipher the reasoning behind the answer or why they gave that answer.” In particular, students are concerned about how instructors would react if something went wrong because they trusted the AI. S11 expects that “I can see a lot of my fellow engineering students finding more room to argue for their marks. I can see people not being as willing to accept their fate with this kind of system.”

*Instructors predicted conflicts between students and the instructor due to AI-based misunderstandings or misleadingness*. For example, a conflict could arise from potential discrepancies between answers from AI, the instructor, and human TAs. As expressed by I4, “Students will argue that, oh AI is wrong. I demand a better assessment right? So, you can say that easily for the AI. But for the authoritative figure like TA and instructor, maybe it's hard to do that.” Similarly, I6 argued a conflict could stem from the opposite direction: “If an AI gives students a great suggestion, if the instructor and TA decided to regrade, it would just be a lot of trouble.” Several instructors (five out of 11) also worried about conflicts that could arise from the quality of response. I1 said that “The concern is the quality of the response, given that there can be ambiguity in the way the students post questions. My concern here is that the algorithm may respond incorrectly or obliquely.” I8 also cautioned AI-based misunderstandings or misleadingness: “If you have a conversation in person, you can always clarify misunderstandings or things like that. I don't think a machine can do that yet. So there's a bit of a potential for misunderstandings so misleading the students.”

### Support

In online learning environments, support refers to the instructor’s instructional management for students, such as providing feedback, explanations, or recommendations directly related to what is being taught (Kang & Im, 2013). Students and instructors expect a positive impact from AI systems in terms of enabling just-in-time personalized support for students at scale, but they expect a negative impact in that excessive support could reduce student agency and ownership of learning.

#### Just-in-time support

*Students believe that AI would support personalized learning experiences, particularly with studying and group projects*. Ultimately, all 12 students felt that AI could help them work to their strengths, mainly in scenarios regarding instructor-independent activities like studying (Scenario 5, 6, 8) and group projects (Scenario 7). Students like S2, S3, and S9 focused on how adaptive technologies could make studying more effective and efficient, as it would “allow [them] to fully understand the concept of what [they’re] learning,” and “allows for them to try and focus on where they might be weaker.” In some cases, the sense of personalization led students to describe the systems as if they could fulfill roles as members of the course team. For example, S1 referred to the Adaptive Quiz system (Scenario 8) as a potential source of guidance: “I think being able to have that quiz to help me, guide me, I’m assuming it would help me.” Likewise, S5 described the presence of an AI Group Project Organizer (Scenario 7) as “having a mentor with you, helping you do it” which would help students “focus more on maybe just researching things, writing their papers, whatever they need to do for the project.”

*Instructors believe AI could be effectively leveraged to help students receive just-in-time personalized support*. I1 said that “one of the best learning mechanisms is to be confronted right away with the correct answer or the correct way of finding the right answer” when doing quizzes and assignments. Many instructors (10 out of 11) expressed approval towards AI-based Intelligent Suggestions (Scenario 5) and an Adaptive Quiz system (Scenario 8). All 11 instructors appreciated how immediate feedback afforded by AI could help students study and effectively understand gaps in their knowledge, particularly at times when they would be unavailable. Similarly, I4 and I11 appreciated that AI could support students who would otherwise be learning asynchronously. For example, AI systems could be supportive of student engagement “because the students are getting real-time answers, particularly in an online world where they may not be in the same time zone, this is a synchronous type [of] learning event for them where they could be doing it when they're studying” (I11).

#### Agency

*Despite the fact that students appreciated the support that they could potentially receive from AI, students perceived that canned and standardized support might have a negative influence on their ability to learn effectively*. For example, S11 shared how he felt the usage of systems that collect engagement data would “over standardize” the learning process by prescribing how an engaged student would or should act. He likened some of the AI examples to “helicopter parenting,” expressing that guidance—whether from an AI or parent—can set an arbitrary pace for a student to follow, despite the fact that the learning experience should involve “learning about yourself and going at your own pace.” Several other students (four out of 12) were concerned with the potential effect of a system like the AI Group Project Organizer (Scenario 7), citing concerns that students “wouldn’t put that much effort” into their group projects because “it might just end up AI doing all the work for them” (S2). Similarly, S6 focused on how AI could detract from the fact that experiences with schoolwork can help students later in life: “… I think it’s like giving them a false sense of security in the sense that like, I’m so used to doing projects with this AI helper that when I go into the real world, I’m not going to be ready. I’m just not going to be prepared for it.”

*Instructors are similarly wary of the fact that too much support from AI could take away students' opportunities for exploration and discovery*. Many instructors (nine out of 11) were concerned that students could lose opportunities to learn new skills or learn from their mistakes. Responding about the AI Group Project Organizer (Scenario 7), I7 stressed that she wouldn’t want to standardize inconsistent group projects since part of an instructor’s job is “helping people understand how group work is conducted… [and] if you’re just laying on a simple answer, you miss that opportunity.” Similarly, other instructors (five out of 12)—primarily those in humanities-based fields—were concerned “it may take the creativity away from the students” since students’ projects “can be hugely different from each other, yet equally good,” and suggestions based on historical data could steer students towards certain directions (I6). I4 even expressed that he currently tries “not to share previous work because [he] thinks that restricts their creativity.” After experiencing all the storyboards related to AI-powered support, I11 presented a vital question: “At what stage is it students’ work and what stage is it the AI’s algorithm?”.

### Presence

In online learning environments, presence refers to a factor that makes students and instructors perceive each other’s existence during the learning process (Kang & Im, 2013). Students and instructors expect the impact of AI systems to be positive in terms of giving them a feeling of improved connectivity, and to be negative in terms of increasing the risk of surveillance problems.

#### Connection

*Students believe that AI can address privacy concerns and support learner–instructor connections by providing social interaction cues without personal camera information*. Many students (10 out of 12) stated that they don’t want to turn on their camera in online courses, even though turning off the camera adversely affects their presence in class, because they have concerns like: “looking like a mess” (S1), “just in my pajamas” (S2), and “feeling too invasive” (S4). Specifically, S9 stated that turning on the camera “makes you more anxious and conscious of what you’re doing and as a result, it deters from you engaging with the content.” In this sense, most students (11 out of 12) liked the Virtual Avatar system (Scenario 9), where AI communicates student facial expressions and body language to the instructor via a virtual avatar. Students expect that this will make them “feel more comfortable going to lecture” (S2), “feel less intrusive for at home learning” (S4), and “showcase much more of their expression or confusion or understanding” (S10). Overall, many students (nine out of 12) appreciated the potential of AI systems as “it solves the problem of not needing to show your actual face, but you can still get your emotions across to the instructor” (S10).

*Instructors believe that the addition of AI would help them become more aware of students’ needs*. Many instructors (10 out of 11), particularly those that taught larger undergraduate courses, stated that students tend to turn off their cameras in online learning spaces, “so something that you really, really miss from teaching online is reading body language” (I10). Instructors generally expressed that AI systems like the Virtual Avatar (Scenario 9) and the AI Facial Analytics (Scenario 11) could be helpful, due to the fact that they would allow students to share their body language and facial expressions without directly sharing their video feed. I4 appreciated that the AI Facial Analytics could automate the process of looking at students’ faces “to see if they got it.” Similarly, I5 liked that a Virtual Avatar could give “any sign that someone is listening,” as “it’s sometimes very tough, especially if [she’s] making a joke.” Furthermore, I4 emphasized that turning on the camera can be helpful not just for the instructor but also for students’ own accountability since “if students don’t turn on the camera, it’s very likely that they are going to do something else.” Overall, instructors appreciated AI’s ability to provide critical information to understand how students are doing and how they feel in online courses.

#### Surveillance

*Although AI can strengthen the connection between students and instructors, students are uncomfortable with the measurement of their unconscious behavior, such as eye tracking or facial expression analysis, because it feels like surveillance*. All 12 students discussed how they would be anxious about being represented by unconscious eye-tracking data. S1 professed: “I don't really know what my eyes are doing. I think it might just make me a little nervous when it comes to taking quizzes or tests and all that. I might be scared that I might have accidentally cheated.” S12 additionally spoke on how that would make her more anxious when sending emails or asking questions due to concern that instructors would judge him based on his unconscious behavior before taking care of his questions. Note that most students (10 out of 12) feel uncomfortable with AI Facial Analytics (Scenario 11). For example, S6 was concerned that facial expression is “something that happens [that] might be outside of your control,” so AI might miss the nuance of authentic human emotion and flatten and simplify it in a way that might cause more confusion. In a similar vein, S11 said that “The nuances of social interaction is something that should be left up to humans and not guided because it’s innately something that, that’s what makes us human is the social interaction portion.” Overall, students complained that they didn’t want to use AI’s measures of unconscious behavior, such as eye tracking or facial expression analysis, even if there are positive aspects.

*Instructors were negative about relying on AI interpretation to understand students’ social interaction cues*. All instructors felt uncomfortable with collecting private data, such as eye movements and facial expressions of students through AI, because “not all the students feel comfortable sharing their private information with the instructor” (I2, I5). Additionally, I9 was concerned that AI Facial Analytics might force students to smile to get a good engagement score, which could adversely affect online learning itself. In this sense, many instructors (nine out of 11) declined to use AI systems that use eye tracking and facial expression analysis in their online courses. Furthermore, I6 would rather “choose to rely on my own kind of sense of the classroom dynamic instead of AI systems” because she believed that the social relationship between students and instructors should be authentic. Plus, other instructors stated they “don’t have time to check all of the interface[s]”, or would have trouble “knowing that that data is accurately reflecting, [that] the student is responding to [their] content” rather than extraneous stimulation in their personal environments (I3, I7). Overall, instructors were uncomfortable with AI giving detailed information about how students engage with their online courses, and they wanted to understand these social interaction cues for themselves.

In summary, students and instructors expect that AI systems will benefit learner–instructor interaction in online learning in terms of improving the quantity and quality of communication, enabling just-in-time personalized support for students at scale, and giving them a feeling of improved connectivity. However, at the same time, students and instructors were concerned that AI systems could create responsibility, agency, and surveillance issues in online learning if they violated social boundaries. These boundaries that make AI perceived to be negative will be discussed in the next section.

## Discussion and conclusion

Our research question focused on examining how students and instructors perceive the impact of AI systems on learner–instructor interaction (inter alia, communication, support, and presence) in online learning. Although the growing body of AIEd research has been conducted to investigate the useful functionalities of AI systems (Seo et al., 2020b; Popenici & Kerr, 2017; Zawacki-Richter et al., 2019), little has been done to understand students’ and instructors’ concerns on AI systems. Recent use of AI systems in online learning showed that careless application can cause surveillance and privacy issues (Lee, 2020), which makes students feel uncomfortable (Bajaj & Li, 2020). In this study, we found that students and instructors perceive the impact of AI systems as double-edged swords. Consequently, although AI systems have been positively recognized for improving the quantity and quality of communication, for providing just-in-time, personalized support for large-scale students, and for improving the feeling of connection, there were concerns about responsibility, agency, and surveillance issues. In fact, what students and instructors perceive negatively often stemmed from the positive aspects of AI systems. For example, students and instructors appreciated AI’s immediate communication, but at the same time they were concerned about AI-based misunderstandings or misleadingness. Although students and instructors valued the just-in-time, personalized support of AI, they feared that AI would limit their ability to learn independently. Students and instructors valued the social interaction cues provided by AI, but they are uncomfortable with the loss of privacy due to AI’s excessive data collection. As shown in Table 6, this study provides rich opportunities to identify the boundaries beyond which AI systems are perceived as “invasive.”

**Table 6:** Summary of the boundaries beyond which AI systems are perceived as invasive, and their potential solutions

Factor of learner–instructor interaction	Boundary beyond which AI systems are perceived as invasive	Potential solution
Communication	Responsibility issues that could arise when AI driven decisions lead to negative consequences	Human-understandable justifications for the AI’s output or procedures (i.e., explainability)
Support	Over-standardizing the learning process by prescribing how an engaged student should act	Bring students and instructors into the decision-making loop and try to inform them of the decision-making context (i.e., human-in-the-loop); make decisions flexible, support multiple paths to success; be more careful about high-stakes decisions
Presence	Uncomfortable with the measurement of their unconscious behavior, such as facial expression analysis or eye tracking, as it feels like surveillance	Establishing clear, simple, and transparent data norms about the nature of data being collected from students and what kind of data is okay to be presented to instructors; maintain agency and provide an effective process of consent for data sharing

First, although AI systems improve instructional communication due to the anonymity it can provide for students, students were concerned about responsibility issues that could arise when AI’s unreliable and unexplained answers lead to negative consequences. For instance, when communicating with an AI Teaching Assistant, the black-box nature of the AI system leaves no choices for students to check whether the answers from AI are right or wrong (Castelvecchi, 2016). Accordingly, students believe they would have a hard time deciphering the reasoning behind an AI’s answer. This can result in serious responsibility issues if students apply an AI’s answers to their tests but instructors mark them as wrong. As well, students would find more room to argue for their marks because of AI’s unreliability.

Acknowledging that AI systems cannot always provide the right answer, a potential solution to this problem is to ensure the system is explainable. Explainability refers to the ability to offer human-understandable justifications for the AI’s output or procedures (Gunning, 2017). Explainability gives students the opportunity to check whether an AI’s answer is right or wrong by themselves, and in doing so can make AI more reliable and responsible (Gunning, 2017). Explainability should be the boundary that determines students’ trust and acceptance of AI systems. How to ensure the explainability of AI systems in the online learning communication context will be an interesting research topic. For example, instead of providing unreliable answers that may mislead or confuse students, AI systems should connect students to relevant sources of information that students can navigate on their own.

Second, while AI systems enable some degree of personalized support, there is a risk of over-standardizing the learning process by prescribing how an engaged student would or should act. Despite the fact that students appreciate the support that they could potentially receive from AI systems, students also worry that canned and standardized support would have a negative influence on their agency over their own learning. Instructors are similarly wary of the fact that too much support from AI systems could take away students’ opportunities for exploration and discovery. Many instructors were concerned that students could lose opportunities to learn new skills or learn from their mistakes.

A solution to mediate this challenge may be to keep instructors involved. The role of AI systems in online education should not be to reduce learning to a set of canned and standardized procedures that reduce the student agency, but rather to enhance human thinking and augment the learning process. In practice, adaptive support is often jointly enacted by AI systems and human facilitators, such as instructors or peers (Holstein et al., 2020). In this context, Baker (2016, p. 603) tried to reconcile humans with AI systems by combining “stupid tutoring systems and intelligent humans.” AI systems can process large amounts of information quickly, but do not respond well to complex contexts. Humans cannot process information as AI systems do, but instead they are flexible and intelligent in a variety of contexts. When AI systems bring human beings into the decision-making loop and try to inform them, humans can learn more efficiently and effectively (Baker, 2016). The human-in-the-loop is the solution to ensure students’ perceived agency in online learning. How to balance artificial and human intelligences to promote students’ agency is an important research direction (e.g., goldilocks conditions for human–AI interaction; Seo et al., 2020a).

Third, even though AI strengthens the perceived connection between students and instructors, students are uncomfortable with the measurement of their unconscious behavior, such as facial expression analysis or eye tracking, as it feels like surveillance. While most students liked the Virtual Avatar system (Scenario 9) where AI simply delivers student facial expressions and body language to the instructor via an avatar, students declined to use the AI Facial Analytics (Scenario 11), which might miss the nuance of social interaction by flattening and simplifying it in a way that might cause more confusion. Interpreting social interaction from unconscious behavior could be the boundary beyond which AI systems are perceived as “invasive.” Students felt uncomfortable about being represented by their unconscious behavior because they did not know what their gaze or face was doing. Stark (2019) described facial recognition as the plutonium of AI: “[facial recognition] is dangerous, racializing, and has few legitimate uses; facial recognition needs regulation and control on par with nuclear waste.” Students complained about their presence being represented by the interpretation of the AI system. In a similar vein, the instructor negatively felt the AI system’s involvement in interpreting the meaning of student behavior.

Establishing clear, simple, and transparent data norms and agreements about the nature of data being collected from students and what kind of data is okay to be presented to instructors are important considerations for future research (Ferguson, 2019; Tsai et al., 2020).

While this study revealed important findings and implications for using AI systems in online learning, the study recognizes some limitations that should be considered when interpreting the patterns of the results. First, although this study attempted to capture various forms of AI systems in online learning based on the ideations from HCI designers and AI experts, it might be possible that other kinds of AI systems exist. Different AI systems might offer different insights. As such, further studies can be conducted with different kinds of AI systems. Next, students’ and instructors’ perceptions of AI systems could be affected by different disciplines. In the current study, we recruited students and instructors in diverse majors and subjects. Although this helped us to generalize our findings from participants with diverse backgrounds, there’s more room to investigate how students and instructors in different disciplines perceive AI systems differently. In our findings, we anecdotally found that instructors in humanities-based fields were more concerned about rapport with students and students’ creativity in courses compared to other disciplines. In order to fully investigate this, future research should consider the different learner–instructor interaction needs of participants from different majors (e.g., engineering vs. humanities).

Another limitation is that the study was conducted by reading the storyboards, rather than directly interacting with AI systems. This might have limited participants’ perceptions about the AI systems. If participants have continuous, direct interactions with the AI systems in the real world, their perceptions may change. As such, future researchers should examine students’ responses to direct exposures of AI systems. This can be accomplished in a variety of ways. For example, one could conduct a lab experiment using virtual reality, the wizard-of-oz method, or the user enactment method to see how students actually respond to AI systems. It would also be meaningful to conduct a longitudinal study to understand whether and/or how student perceptions would change over time.

### Theoretical implications

This study provides theoretical implications for a learner–instructor interaction framework by highlighting and mapping key challenges in AI-related ethical issues (i.e. responsibility, agency, and surveillance) in online learning environments. Researchers have requested clear ethical guidelines for future research to prevent AI systems from accidently harming people (Loi et al., 2019). Although several ethical frameworks and professional codes of conduct have been developed to mitigate the potential dangers and risks of AI in education, significant debates continue about their specific impact on students and instructors (Williamson & Eynon, 2020). The results of this study increase our understanding of the boundaries that determine student and instructor trust and acceptance of AI systems, and provide a theoretical background for designing AI systems that positively support learner–instructor interactions in a variety of learning situations.

### Practical implications

This study has practical implications for both students and instructors. Interestingly, most of the negative experiences with AI systems came from students’ unrealistic expectations and misunderstandings about AI systems. The AI system’s answer is nothing more than an algorithm based on accumulated data, yet students typically expect the AI system to be accurate. These misconceptions can be barriers to the effective use of AI systems by students and instructors. To address this, it is important to foster AI literacy in students and instructors without a technical background (Long & Magerko, 2020). For example, recent studies have published guides on how to incorporate AI into K-12 curricula (Touretzky et al., 2019), and researchers are exploring how to engage young learners in creative programming activities involving AI (Zimmermann-Niefield et al., 2019).

Furthermore, in order to minimize the negative impact of AI systems on learner–instructor interaction, it is important to address tensions where AI systems violate the boundaries between students and instructors (e.g., responsibility, agency, and surveillance issues). We proposed that future AI systems should ensure explainability, human-in-the-loop, and careful data collection and presentation. By doing so, AI systems will be more closely integrated into future online learning. It is important to note that the present study does not argue that AI systems will replace the entire role of human instructors. Rather, in the online learning of the future, AI systems and humans will work closely together, and for this, it is important to use these systems with consideration about perceived affordances and drawbacks.

## Data Availability

The full set of storyboards and an example of our codes can be viewed at https://osf.io/3aj5v/?view_only=bc5fa97e6f7d46fdb66872588ff1e22e.

## Appendix A: summary of the AI experts’ information

**Table 7:** Summary of the AI experts’ information

ID	AI research experience	AI research subject	Teaching experience	Teaching subject
1	11 years	Computer graphics, computer vision using machine learning	2 years	Deep learning for computer vision and graphics
2	15 years	Business analytics	5 years	Business analytics
3	24 years	Machine learning, tools for creativity	18 years	Machine learning, computer graphics, AI, HCI
4	3 years	Learning technologies	10 years	Language education
5	8 years	Intelligent transport systems, data science	2 years	Transport planning
6	4 years	Learning sciences, sales enablement	11 years	Organizational behaviour and human resource management

## Appendix B: speed dating interview script

1. Introduction

- Hello, thank you for taking time for this interview today. We’re really looking forward to learning from your experience with online learning.
- Today, we’ll be discussing a set of 11 storyboards that are related to AI systems for online courses. When reading the storyboards, try to think about them in the context of your discipline and experiences. Our goal is to reveal your perceptions of AI in online learning.
- For your information, the interview will take about 60 min. The interview will be audio recorded but will be confidential and de-identified.

2. For each storyboard

- Do you think this AI system supports learner–instructor interaction? Yes, no, or do you feel neutral? Why?
- [When the participant is a student] Would the incorporation of this AI system into your courses change your interaction with the instructor?
- [When the participant is an instructor] Would incorporating this AI system into the course change how you interact with students?
- Do you have any reservations or concerns about this AI system?

3. After examining all storyboards (capturing participants’ holistic point of view)

- Of the storyboards shown today, which AI systems do you think would work well in your online classroom? Why? Also, which ones wouldn’t work well?
- How do you think the adoption of AI would affect the relationship between students and the instructor?

4. Conclusion

- Do you have any final comments?
- Thank you for taking the time to interview with us today. We really appreciate that you took time to participate in our study and share your expertise. Your insights were really helpful.

## References

[CR1] Andersen, J. C. (2013). *Learner satisfaction in online learning: An analysis of the perceived impact of learner-social media and learner–instructor interaction*. Doctoral dissertation. East Tennessee State University, Tennessee.

[CR2] Anderson, J. R., Boyle, C. F., & Reiser, B. J. (1985). Intelligent tutoring systems. *Science,**228*(4698), 456–462. 17746875 10.1126/science.228.4698.456

[CR3] Aslan, S., Alyuz, N., Tanriover, C., Mete, S. E., Okur, E., D'Mello, S. K., & Arslan Esme, A. (2019). Investigating the impact of a real-time, multimodal student engagement analytics technology in authentic classrooms. In: *Proceedings of the 2019 CHI conference on human factors in computing systems* (pp. 1–12).

[CR4] Bajaj, M., & Li, J. (2020). *Students, faculty express concerns about online exam invigilation amidst COVID-19 outbreak*. Retrieved February 8, 2021, from https://www.ubyssey.ca/news/Students-express-concerns-about-online-exams/

[CR5] Baker, R. S. (2016). Stupid tutoring systems, intelligent humans. *International Journal of Artificial Intelligence in Education,**26*(2), 600–614.

[CR6] Banna, J., Lin, M. F. G., Stewart, M., & Fialkowski, M. K. (2015). Interaction matters: Strategies to promote engaged learning in an online introductory nutrition course. *Journal of Online Learning and Teaching/MERLOT,**11*(2), 249. PMC4948751 27441032

[CR7] Beard, A. (2020). *Can computers ever replace the classroom?*. Retrieved January 10, 2021, from https://www.theguardian.com/technology/2020/mar/19/can-computers-ever-replace-the-classroom

[CR15] Braun, V., & Clarke, V. (2006). Using thematic analysis in psychology. *Qualitative Research in Psychology,**3*(2), 77–101.

[CR16] Castelvecchi, D. (2016). Can we open the black box of AI? *Nature News,**538*(7623), 20. 10.1038/538020a 27708329

[CR17] Chan, R. (2019). *The Cambridge Analytica whistleblower explains how the firm used Facebook data to sway elections*. Business Insider. Retrieved from https://www.businessinsider.com/cambridge-analytica-whistleblower-christopher-wylie-facebook-data-2019-10

[CR18] Crawford, K., & Calo, R. (2016). There is a blind spot in AI research. *Nature,**538*(7625), 311–313. 27762391 10.1038/538311a

[CR19] Cruz-Benito, J., Sánchez-Prieto, J. C., Therón, R., & García-Peñalvo, F. J. (2019). Measuring students’ acceptance to AI-driven assessment in eLearning: Proposing a first TAM-based research model. In: *International conference on human–computer interaction* (pp. 15–25). Springer, Cham.

[CR20] Davidoff, S., Lee, M. K., Dey, A. K., & Zimmerman, J. (2007). Rapidly exploring application design through speed dating. In: *International conference on ubiquitous computing* (pp. 429–446). Springer, Berlin, Heidelberg.

[CR21] Felix, C. V. (2020). The role of the teacher and AI in education. In: *International perspectives on the role of technology in humanizing higher education*. Emerald Publishing Limited.

[CR22] Ferguson, R. (2019). Ethical challenges for learning analytics. *Journal of Learning Analytics,**6*(3), 25–30.

[CR11] Fong, M., Dodson, S., Harandi, N. M., Seo, K., Yoon, D., Roll, I., & Fels, S. (2019). Instructors desire student activity, literacy, and video quality analytics to improve video-based blended courses. In *Proceedings of the Sixth (2019) ACM Conference on Learning@ Scale* (pp. 1–10).

[CR23] Goel, A. K., & Polepeddi, L. (2016). *Jill Watson: A virtual teaching assistant for online education*. Georgia Institute of Technology.

[CR24] Guilherme, A. (2019). AI and education: The importance of teacher and student relations. *AI & Society,**34*(1), 47–54.

[CR25] Gunning, D. (2017). Explainable artificial intelligence (xai). Defense Advanced Research Projects Agency (DARPA), *nd Web, 2*(2).

[CR26] Heidicker, P., Langbehn, E., & Steinicke, F. (2017). Influence of avatar appearance on presence in social VR. In: *2017 IEEE symposium on 3D user interfaces (3DUI)* (pp. 233–234). IEEE.

[CR27] Holstein, K., Hong, G., Tegene, M., McLaren, B. M., & Aleven, V. (2018). The classroom as a dashboard: Co-designing wearable cognitive augmentation for K-12 teachers. In: *Proceedings of the 8th international conference on learning analytics and knowledge* (pp. 79–88).

[CR28] Holstein, K., Aleven, V., & Rummel, N. (2020). A conceptual framework for human–AI hybrid adaptivity in education. In: *International conference on artificial intelligence in education* (pp. 240–254). Springer, Cham.

[CR29] Hwang, G. J., Xie, H., Wah, B. W., & Gašević, D. (2020). Vision, challenges, roles and research issues of Artificial Intelligence in Education. *Computers and Education: Artificial Intelligence,**1*, 100001.

[CR30] Jou, M., Lin, Y. T., & Wu, D. W. (2016). Effect of a blended learning environment on student critical thinking and knowledge transformation. *Interactive Learning Environments,**24*(6), 1131–1147.

[CR31] Kang, M. S. (2010). *Development of learners’ perceived interaction model and scale between learner and instructor in e-learning environments*. Doctoral dissertation. Korea University, Korea.

[CR32] Kang, M., & Im, T. (2013). Factors of learner–instructor interaction which predict perceived learning outcomes in online learning environment. *Journal of Computer Assisted Learning,**29*(3), 292–301.

[CR33] Laura, R. S., & Chapman, A. (2009). The technologisation of education: Philosophical reflections on being too plugged in. *International Journal of Children’s Spirituality,**14*(3), 289–298.

[CR34] Lee, S. (2020). *Proctorio CEO releases student’s chat logs, sparking renewed privacy concerns*. Retrieved February 8, 2021, from https://www.ubyssey.ca/news/proctorio-chat-logs/

[CR35] Linsey, J. S., & Becker, B. (2011). Effectiveness of brainwriting techniques: comparing nominal groups to real teams. In: *Design creativity 2010* (pp. 165–171). Springer.

[CR36] Loi, D., Wolf, C. T., Blomberg, J. L., Arar, R., & Brereton, M. (2019). Co-designing AI futures: Integrating AI ethics, social computing, and design. In: *Companion publication of the 2019 on designing interactive systems conference 2019 companion* (pp. 381–384).

[CR37] Long, D., & Magerko, B. (2020). What is AI literacy? Competencies and design considerations. In: *Proceedings of the 2020 CHI conference on human factors in computing systems* (pp. 1–16).

[CR38] Luckin, R. (2017). Towards artificial intelligence-based assessment systems. *Nature Human Behaviour,**1*(3), 1–3.

[CR39] Luo, N., Zhang, M., & Qi, D. (2017). Effects of different interactions on students’ sense of community in e-learning environment. *Computers & Education,**115*, 153–160.

[CR40] Luria, M., Zheng, R., Huffman, B., Huang, S., Zimmerman, J., & Forlizzi, J. (2020). Social boundaries for personal agents in the interpersonal space of the home. In: *Proceedings of the 2020 CHI conference on human factors in computing systems* (pp. 1–12).

[CR41] Martin, F., & Bolliger, D. U. (2018). Engagement matters: Student perceptions on the importance of engagement strategies in the online learning environment. *Online Learning,**22*(1), 205–222.

[CR42] Martin, F., Wang, C., & Sadaf, A. (2018). Student perception of helpfulness of facilitation strategies that enhance instructor presence, connectedness, engagement and learning in online courses. *The Internet and Higher Education,**37*, 52–65.

[CR43] McArthur, A. (2020). *Students struggle with online test proctoring systems*. Retrieved January 10, 2021, from https://universe.byu.edu/2020/12/17/students-struggle-with-online-test-proctoring-systems/

[CR44] Misiejuk, K., & Wasson, B. (2017). *State of the field report on learning analytics*. Centre for the Science of Learning & Technology (SLATE), University of Bergen.

[CR45] Moore, M. G. (1989). Three types of interaction. *American Journal of Distance Education,**3*(2), 1–7.

[CR46] Murphy, R. F. (2019). Artificial intelligence applications to support K–12 teachers and teaching. *RAND Corporation*. 10.7249/PE315

[CR47] Nguyen, T. D., Cannata, M., & Miller, J. (2018). Understanding student behavioral engagement: Importance of student interaction with peers and teachers. *The Journal of Educational Research,**111*(2), 163–174.

[CR48] Nowell, L. S., Norris, J. M., White, D. E., & Moules, N. J. (2017). Thematic analysis: Striving to meet the trustworthiness criteria. *International Journal of Qualitative Methods,**16*(1), 1609406917733847.

[CR49] Perin, D., & Lauterbach, M. (2018). Assessing text-based writing of low-skilled college students. *International Journal of Artificial Intelligence in Education,**28*(1), 56–78.

[CR50] Popenici, S. A., & Kerr, S. (2017). Exploring the impact of artificial intelligence on teaching and learning in higher education. *Research and Practice in Technology Enhanced Learning,**12*(1), 22. 30595727 10.1186/s41039-017-0062-8 PMC6294271

[CR51] Richardson, J. C., Maeda, Y., Lv, J., & Caskurlu, S. (2017). Social presence in relation to students’ satisfaction and learning in the online environment: A meta-analysis. *Computers in Human Behavior,**71*, 402–417.

[CR52] Robinson, H., Kilgore, W., & Warren, S. (2017). Care, communication, support: Core for designing meaningful online collaborative learning. *Online Learning Journal.*10.24059/olj.v21i4.1240

[CR8] Roll, I., & Winne, P. H. (2015). Understanding, evaluating, and supporting self-regulated learning using learning analytics. *Journal of Learning Analytics, 2*(1), 7–12.

[CR9] Roll, I., & Wylie, R. (2016). Evolution and revolution in artificial intelligence in education. *International Journal of Artificial Intelligence in Education, 26*(2), 582–599.

[CR10] Roll, I., Russell, D. M., & Gašević, D. (2018). Learning at scale. *International Journal of Artificial Intelligence in Education, 28*(4), 471–477.

[CR53] Ross, B., Chase, A. M., Robbie, D., Oates, G., & Absalom, Y. (2018). Adaptive quizzes to increase motivation, engagement and learning outcomes in a first year accounting unit. *International Journal of Educational Technology in Higher Education,**15*(1), 30.

[CR12] Seo, K., Fels, S., Kang, M., Jung, C., & Ryu, H. (2020a). Goldilocks conditions for workplace gamification: How narrative persuasion helps manufacturing workers create self-directed behaviors. *Human–Computer Interaction*. 1–38.

[CR13] Seo, K., Fels, S., Yoon, D., Roll, I., Dodson, S., & Fong, M. (2020b). Artificial intelligence for video-based learning at scale. In *Proceedings of the Seventh ACM Conference on Learning@ Scale* (pp. 215–217).

[CR14] Seo, K., Dodson, S., Harandi, N. M., Roberson, N., Fels, S., & Roll, I. (2021). Active learning with online video: The impact of learning context on engagement. *Computers & Education, 165*, 104132.

[CR54] Shackelford, J. L., & Maxwell, M. (2012). Contribution of learner–instructor interaction to sense of community in graduate online education. *MERLOT Journal of Online Learning and Teaching, 8*(4), 248–260.

[CR55] Stark, L. (2019). Facial recognition is the plutonium of AI. *XRDS: Crossroads, the ACM Magazine for Students,**25*(3), 50–55.

[CR56] Touretzky, D., Gardner-McCune, C., Martin, F., & Seehorn, D. (2019). Envisioning AI for K-12: What should every child know about AI?. In: *Proceedings of the AAAI conference on artificial intelligence* (Vol. 33, No. 01, pp. 9795–9799).

[CR57] Truong, K. N., Hayes, G. R., & Abowd, G. D. (2006). Storyboarding: an empirical determination of best practices and effective guidelines. In: *Proceedings of the 6th conference on designing interactive systems* (pp. 12–21).

[CR58] Tsai, Y. S., Whitelock-Wainwright, A., & Gašević, D. (2020). The privacy paradox and its implications for learning analytics. In: *Proceedings of the tenth international conference on learning analytics & knowledge* (pp. 230–239).

[CR59] VanLehn, K. (2011). The relative effectiveness of human tutoring, intelligent tutoring systems, and other tutoring systems. *Educational Psychologist,**46*(4), 197–221.

[CR60] Walker, C. H. (2016). *The correlation between types of instructor-student communication in online graduate courses and student satisfaction levels in the private university setting*. Doctoral dissertation. Carson-Newman University, Tennessee.

[CR61] Williamson, B., & Eynon, R. (2020). Historical threads, missing links, and future directions in AI in education. *Learning, Media and Technology,**45*(3), 223–235.

[CR62] Wogu, I. A. P., Misra, S., Olu-Owolabi, E. F., Assibong, P. A., Udoh, O. D., Ogiri, S. O., & Damasevicius, R. (2018). Artificial intelligence, artificial teachers and the fate of learners in the 21st century education sector: Implications for theory and practice. *International Journal of Pure and Applied Mathematics,**119*(16), 2245–2259.

[CR63] Woolf, B. P., Arroyo, I., Muldner, K., Burleson, W., Cooper, D. G., Dolan, R., & Christopherson, R. M. (2010). The effect of motivational learning companions on low achieving students and students with disabilities. In: *International conference on intelligent tutoring systems* (pp. 327–337). Springer, Berlin, Heidelberg.

[CR64] Zawacki-Richter, O., Marín, V. I., Bond, M., & Gouverneur, F. (2019). Systematic review of research on artificial intelligence applications in higher education–where are the educators? *International Journal of Educational Technology in Higher Education,**16*(1), 39.

[CR65] Zhang, C., Chen, H., & Phang, C. W. (2018). Role of instructors’ forum interactions with students in promoting MOOC continuance. *Journal of Global Information Management (JGIM),**26*(3), 105–120.

[CR66] Zimmerman, J., & Forlizzi, J. (2017). Speed dating: Providing a menu of possible futures. *She Ji: THe Journal of Design, Economics, and Innovation,**3*(1), 30–50.

[CR67] Zimmermann-Niefield, A., Turner, M., Murphy, B., Kane, S. K., & Shapiro, R. B. (2019). Youth learning machine learning through building models of athletic moves. In *Proceedings of the 18th ACM international conference on interaction design and children* (pp. 121–132).

